# Amputation of Fungus Hæmatodes of the Breast

**Published:** 1829-01-01

**Authors:** 


### LXII.

### AMPUTATION OF FUNGUS HÆMATODES OF THE BREAST.

In the Midland Reporter, Mr. Fletcher, surgeon to the Gloucester Infirmary, has published a case of fungus hæmatodes of the breast, which he lately removed by amputation.

The patient was a married lady, aged 35, who consulted Mr. F. in October, 1827, with a tumour in the breast, of considerable size. She had first perceived it, when the size of a walnut, ten years before, but it gave her no pain, and did not perceptibly increase, till the Spring of the year 1825, when she weaned her second child. The tumour was the seat of dull heavy pain, and was subject to periodical attacks of inflammation, when the axillary glands became temporarily swollen. Mr. Fletcher recommended amputation, but the patient would not consent. Iodine and a course of mercury, with several other means were tried without benefit, and in July, 1828, the lady submitted to the removal of the disease by the knife. At this time the size of the tumour was great, and it seemed to consist of several masses, having a firm and somewhat elastic feel. The venous ramifications were numerous and large—the patient complained of a dull heavy pain—there was slight adhesion near the sterno-clavicular articulation,—and the glands of the neighbourhood were not affected. Mr. Fletcher considered it fungus hæmatodes, and prepared for much bleeding during the operation. The steps of this we need not describe, suffice it to say, that such bleeding took place as reduced the powers to a very low ebb, from which depressed state the lady, however, recovered. During the first five weeks after the operation, small vascular spots, with yellow spots deposited around them, were constantly presenting themselves in different parts of the surface of the wound. This having been purposely kept open, these morbid depositions were instantly destroyed by the strong arsenical ointment, or nitric acid slightly diluted. At the date of report, the wound had a healthy granulating surface and was contracted to the dimension of a crown-piece. The patient presents, says the report, a prospect most favourable and satisfactory, to which, we wish we could say Amen !

The cases of fungus hæmatodes in which we have seen an operation performed, have, in almost every instance, ended ill. This, however, is nothing in the scale of testimony, for sorry we are to add, that a gentleman who has necessarily seen a great deal of the disease, Mr. Brodie, entertains but a gloomy opinion of the ultimate results of amputation. Mr. B., in his Clinical Lectures, observes, that patients who have undergone an operation for fungus hæmatodes, are liable to return of the disease in the lungs. This fungus hæmatodes of the pulmonary structure is extremely insidious in its progress, and is mostly attended with few or no symptoms to mark its existence. The individual, however, takes cold, and suddenly symptoms of effusion into the cavity of the chest, come on and rapidly destroy him, when dissection discovers the real state of things, fungus hæmatodes of the lung.

---

